# A Comprehensive Approach to QTc Interval in Rheumatoid Arthritis

**DOI:** 10.5152/ArchRheumatol.2026.11056

**Published:** 2026-03-03

**Authors:** Yusuf Ziya Şener, Seher Sener

**Affiliations:** 1 Department of Cardiology, Erasmus MC Thoraxcenter, Rotterdam, The Netherlands; 2 Division of Pediatric Rheumatology, Department of Pediatrics, Erasmus MC, Rotterdam, The Netherlands

To the Editor,

We have read the article published by Gulkesen et al^1^ with great interest. The authors compared the electrocardiographic (ECG) parameters suggestive of arrhythmic risk between patients with rheumatoid arthritis (RA) and a control group of healthy individuals. Electrocardiographic parameters, including corrected QT (QTc) interval, QT dispersion (QTd), P wave, P wave dispersion, Tp-e interval (the interval from the peak to the end of the T wave), and Tp-e/QTc ratio, were significantly different among the groups, indicating increased risk of arrhythmia in patients with RA.^1^ We believe that these findings are valuable in demonstrating the importance of ECG assessment in cases with RA. Building on these findings, this letter aims to highlight additional factors, such as hydroxychloroquine (HCQ) use and electrolyte disturbances, that may influence the QTc interval and arrhythmic risk in RA patients.

Hydroxychloroquine is an antimalarial drug that is also used in the treatment of several rheumatic diseases as a disease-modifying antirheumatic drug (DMARD). Despite the lack of recommendation for the use of HCQ as a monotherapy in RA, evidence suggests that the addition of HCQ to other DMARDs may improve clinical outcomes in patients with RA.^2^ Previous studies established that the use of HCQ is associated with a moderate increase in QTc interval.^3^ It can be postulated that the administration of HCQ may have contributed to the observed increase in the QTc interval in patients with RA, so assessment of HCQ use would be valuable.

Hypokalemia results in alterations to the ECG findings, leading to a prolongation of the QTc interval and the appearance of U waves.^4^ Distal renal tubular acidosis (dRTA) develops as a result of failure to acidify the urine in the distal parts of the nephron, and hypokalemia is a characteristic finding of dRTA.^5^ It may occur during the course of rheumatic diseases, including RA, particularly when accompanied by Sjögren’s syndrome.^6^ Moreover, steroids may also induce hypokalemia.^7^ Given that steroids are used in the treatment of RA and may potentially lead to the development of dRTA in patients with RA, it would be prudent to evaluate the potassium level and its impact on the QTc interval.

In conclusion, a number of factors, including the use of certain medications and electrolyte imbalances, have been demonstrated to exert a considerable influence on the QTc interval and the propensity for arrhythmic events.^8,9^ Consequently, these factors should also be taken into account in the evaluation of ECG parameters and the assessment of arrhythmic risk.

## Data Availability

The data that support the findings of this study are available on request from the corresponding author.
